# Measuring Covid-related Ageism

**DOI:** 10.1093/geroni/igab046.3625

**Published:** 2021-12-17

**Authors:** Amanda Sokan, Mindy Fain, Jake Harwood, Kathleen Insel, Zhao Chen, Linda Phillips

**Affiliations:** 1 The University of Arizona, University of Arizona, Arizona, United States; 2 University of Arizona Center on Aging, Tucson, Arizona, United States; 3 University of Arizona, University of Arizona, Arizona, United States; 4 University of Arizona, Tucson, Arizona, United States; 5 The University of Arizona, Tucson, Arizona, United States; 6 College of Nursing, University of Arizona, Arizona, United States

## Abstract

Prejudice, discrimination, and negative stereotypes based on age (ageism) are long-standing and strongly implicated in poor health outcomes and limited access to health care for older adults. Recent writings suggest the COVID-19 pandemic raised the specter of ageism to an entirely new level. Do these observations reflect an exaggeration of “usual” ageism or a unique manifestation of intergenerational tension rooted in resentments of younger people concerning COVID-related disruptions in their lives believed to be primarily a function of older people’s vulnerability to the disease phenomenon? To address this question, the purpose of this study was to develop and test an instrument to measure ageist tendencies associated with the COVID-19 pandemic. Scale items, written to reflect attitudes about paternalism, inconvenience, and sacrifice, were assessed for content validity. Then the 12-item scale was administered to 227 undergraduate and graduate students in the health and social sciences. Analysis showed items have strong internal consistency and concurrent and discriminant validity. Importantly the scale explained unique variance over and above other standard measures of ageism. Ageism is deeply embedded in global and U.S. culture and strongly related to negative outcomes. This scale will assist researchers investigating the ageist consequences of the current pandemic and help us to monitor what could be long-term residual ageist effects of the COVID pandemic.

